# Global research landscape of ferroptosis in colorectal cancer: a multi-database bibliometric analysis (2017–2025)

**DOI:** 10.3389/fonc.2026.1918936

**Published:** 2026-10-02

**Authors:** Fengfeng You, Rong Tan, Fengxi Long, Bing Yang, Dongxin Tang

**Affiliations:** 1 The First College of Clinical Medicine, Guizhou University of Traditional Chinese Medicine, Guiyang, China; 2 Department of Pharmaceutics, The First Affiliated Hospital of Guizhou University of Traditional Chinese Medicine, Guiyang, China; 3 The First Affiliated Hospital of Guizhou University of Traditional Chinese Medicine, Guiyang, China; 4 Student Management Office, The First Affiliated Hospital of Guizhou University of Traditional Chinese Medicine, Guiyang, China; 5 Department of Oncology, The First Affiliated Hospital of Guizhou University of Traditional Chinese Medicine, Guiyang, China

**Keywords:** bibliometric analysis, colorectal cancer, ferroptosis, research trends, tumor immune microenvironment

## Abstract

**Objective:**

Ferroptosis, an iron-dependent form of regulated cell death, has attracted increasing attention in recent years because of its critical roles in tumor progression, therapeutic resistance, and immune regulation. Colorectal cancer (CRC) is one of the most prevalent malignancies worldwide and imposes a substantial mortality burden. This study aimed to systematically analyze the global research landscape, hotspots, and developmental trends of ferroptosis in CRC research from 2017 to 2025.

**Methods:**

English-language publications related to ferroptosis and CRC published between January 1, 2017, and December 31, 2025, were retrieved from the Web of Science Core Collection (WoSCC) and Scopus databases. A total of 1, 354 original articles and reviews were included. VOSviewer, CiteSpace, and the bibliometrix package in R were used for bibliometric and visualization analyses to evaluate annual publication trends, country/region and institutional collaboration networks, author contributions, journal distribution, co-cited references, keyword co-occurrence, and thematic evolution.

**Results:**

The number of publications in this field increased rapidly after 2020 and peaked in 2025. China ranked first in both publication output and total citations, followed by the United States, and China–United States collaboration constituted the core of the international collaboration network. Central South University was the most productive institution, while journals such as Frontiers in Oncology and Frontiers in Immunology served as major publication platforms. Research hotspots were mainly concentrated on chemotherapy resistance, the tumor immune microenvironment, PANoptosis, prognostic models, non-coding RNAs, gut microbiota, and the regulation of ferroptosis by traditional Chinese medicine and natural products.

**Conclusion:**

Ferroptosis has become a rapidly evolving frontier in CRC research. The research focus is gradually shifting from fundamental molecular mechanisms toward translational medical directions, including chemosensitization, combination immunotherapy, reversal of drug resistance, and precise prognostic evaluation. This study provides a systematic reference for understanding the global knowledge structure and future research directions in the field of ferroptosis and CRC.

## 1 Introduction

Colorectal cancer (CRC) represents a major global health challenge, ranking as the third most common cancer worldwide and the second leading cause of cancer-related death globally (1). The increasing incidence of CRC is closely associated with modifiable lifestyle factors, including smoking, alcohol consumption, dietary patterns rich in red and processed meats, obesity, and physical inactivity (2). Approximately 70%–90% of CRC cases are classified as adenocarcinoma, and their initiation and progression typically begin with mutations in the tumor suppressor gene adenomatous polyposis coli (APC), followed by activation of the oncogene Kirsten rat sarcoma viral oncogene homolog (KRAS), thereby driving adenoma progression and ultimately leading to TP53 inactivation, which promotes malignant transformation (3, 4). These mutations disrupt key signaling pathways, gene expression, and metabolic homeostasis, conferring proliferative and survival advantages on malignant cells. Current treatment modalities include surgical resection, neoadjuvant radiotherapy, palliative chemotherapy, and emerging approaches such as targeted therapy and immunotherapy (5). Recent studies have identified ferroptosis-related proteins as highly promising therapeutic targets, and multiple ferroptosis inducers have shown efficacy in CRC research (6). These findings reveal a novel strategy for treating CRC through modulation of ferroptosis.

Ferroptosis is an iron-dependent form of cell death involving free radical and lipid metabolism, resulting in iron-dependent lipid peroxidation and ultimately cell death (7). The core mechanisms of ferroptosis revolve around three interconnected processes (**Figure 1**): dysregulated iron homeostasis, excessive lipid peroxidation, and impaired antioxidant defense (8). Its prominent biochemical features include depletion of glutathione (GSH) and inactivation of glutathione peroxidase 4 (GPX4), resulting in the failure of lipid peroxides to be metabolized through the GPX4-catalyzed GSH-dependent reduction reaction; Fe²^+^ subsequently oxidizes lipids to generate reactive oxygen species (ROS), thereby promoting the occurrence of ferroptosis (9). Studies have shown that ferroptosis plays a role in multiple cancers, including hepatocellular carcinoma, lung cancer, breast cancer, CRC, pancreatic ductal adenocarcinoma, and prostate cancer (10). During oncogenesis, systemic and cellular iron metabolism undergoes substantial reprogramming, thereby promoting malignant progression. As an intrinsic tumor-suppressive mechanism, ferroptosis participates in tumor biological processes, influences cancer cell metabolism, the immune microenvironment, and therapeutic sensitivity, and has emerged as a potential therapeutic target for overcoming cancer initiation, progression, and drug resistance (11, 12). Studies have found that ferroptosis can also regulate T-cell function, enabling T cells to exert immune effects in the recognition and clearance of cancer cells (13, 14). Specifically, in CRC, induction of ferroptosis through inhibition of SLC7A11 or inactivation of GPX4 demonstrates therapeutic potential by eliminating apoptosis-resistant malignant cells (15). Meanwhile, ferroptosis inducers can enhance the sensitivity of CRC cells to chemotherapeutic agents, improve the tumor immune microenvironment, and activate antitumor immune responses (16). Although clinical translation remains at the investigative stage, ferroptosis-based interventions represent a rapidly evolving frontier in CRC therapy.

**Figure 1:**
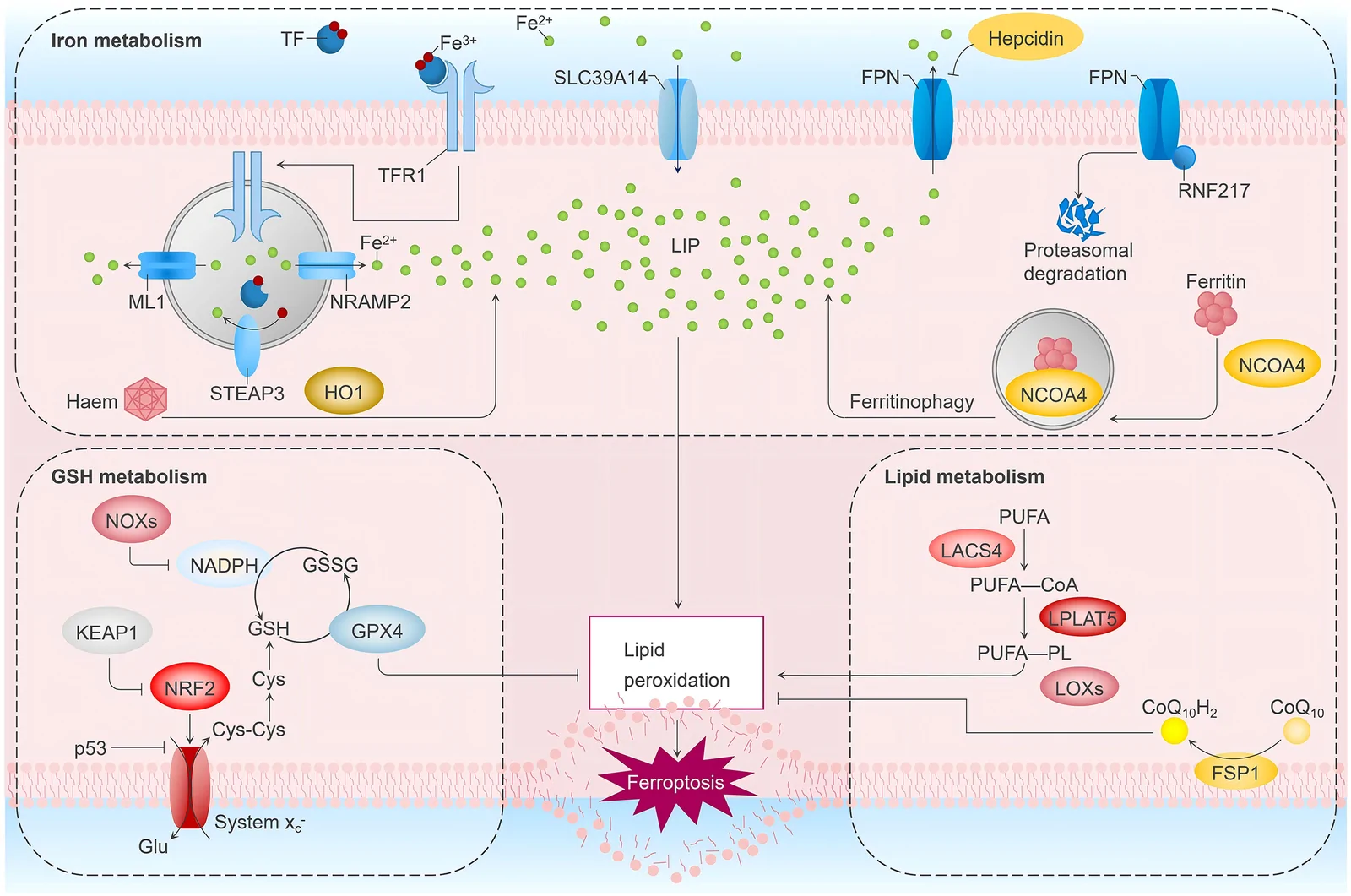
Core mechanisms of ferroptosis.

Notably, ferroptosis has emerged as an immunogenic cell death mechanism complementary to conventional immunotherapy, and targeted induction of ferroptosis is increasingly recognized as a promising strategy for cancer treatment (17). Although the number of publications addressing the association between ferroptosis and CRC continues to increase, a systematic bibliometric evaluation of this interdisciplinary connection remains lacking. Bibliometric analysis is a methodological framework used to quantitatively assess temporal trends, knowledge structures, and disciplinary impact within a scientific field, enabling systematic mapping of developmental trajectories and emerging research priorities (18). Currently, bibliometric studies on ferroptosis in cancer have mainly focused on individual malignancies in therapeutic contexts, such as hepatocellular carcinoma (19), lung cancer (20), and gastric cancer (21). In the present study, we performed a bibliometric analysis of publications related to ferroptosis in CRC from 2017 to 2025 by mapping temporal publication trends and collaboration networks among countries, institutions, and authors, identifying high-impact journals and seminal works, and visualizing keyword clustering patterns and thematic evolution, with the aim of identifying future directions for therapeutic development in this field.

## 2 Materials and methods

### 2.1 Data sources and search strategy

The data used in this study were obtained from the Web of Science Core Collection (WoSCC) and Scopus databases, which are regarded as two of the most widely used resources for bibliometric analysis (22). Keyword selection was based on a broad definition of the field and related terminology, encompassing commonly used medical terms, Medical Subject Headings (MeSH), and disease-related synonyms. The retrieval period ranged from January 1, 2017, to December 31, 2025. To minimize data discrepancies resulting from database updates, all searches and data exports were performed on August 12, 2026.

The advanced search function was applied using the following strategy: (“Colorectal Neoplasm*” OR “Colorectal Tumor*” OR “Colorectal Cancer*” OR “Colorectal Carcinoma*” OR “Colonic Neoplasm*” OR “Colon Neoplasm*” OR “Cancer of Colon” OR “Colon Cancer*” OR “Colon Carcinoma*” OR “Colonic Cancer*” OR “Rectal Neoplasm*” OR “Rectal Tumor*” OR “Rectal Cancer*” OR “Rectal Carcinoma*” OR “Rectum Neoplasm*” OR “Rectum Cancer*” OR “Cancer of Rectum” OR “CRC” OR “Colorectal Adenocarcinoma*”) AND “Ferroptosis”. Because WoSCC and Scopus use different indexing structures, database-specific search fields were applied. The Topic (TS) field was selected in the WoSCC database, and the TITLE-ABS-KEY field was selected in the Scopus database. Although differences in indexing between the databases may result in non-identical sets of retrieved records, the combined use of both databases was intended to improve overall literature coverage and compensate for the limitations of relying on a single database. Therefore, the inherent differences between the indexing and retrieval systems of these two databases were fully considered.

### 2.2 Data selection and standardization

Based on the search strategy, 1, 119 records were retrieved and downloaded from WoSCC, and 1, 354 records were retrieved and downloaded from Scopus. All records retrieved from the two databases were imported into NoteExpress for subsequent management, screening, and classification. First, records from the two databases were merged, and duplicate publications were removed based on article titles and DOI numbers. The publications were then screened according to the inclusion and exclusion criteria. The inclusion criteria were as follows (1): publication type: original research articles and review articles; and (2) language: publications written in English. The exclusion criteria were as follows (1): non-English publications (2); publication types including corrections, meeting abstracts, editorial materials, book chapters, letters, and notes; and (3) publications unrelated to ferroptosis and CRC. The literature screening process (**Figure 2**) was conducted independently by two researchers (Ff.Y. and R.T.). Inter-rater agreement was assessed using Cohen’s kappa coefficient (κ = 0.85), indicating a high level of agreement between the two researchers. Any disagreements were reviewed and resolved with the involvement of a third researcher (Fx.L.). Ultimately, 1, 354 eligible publications were included in the bibliometric analysis of the association between ferroptosis and CRC.

**Figure 2:**
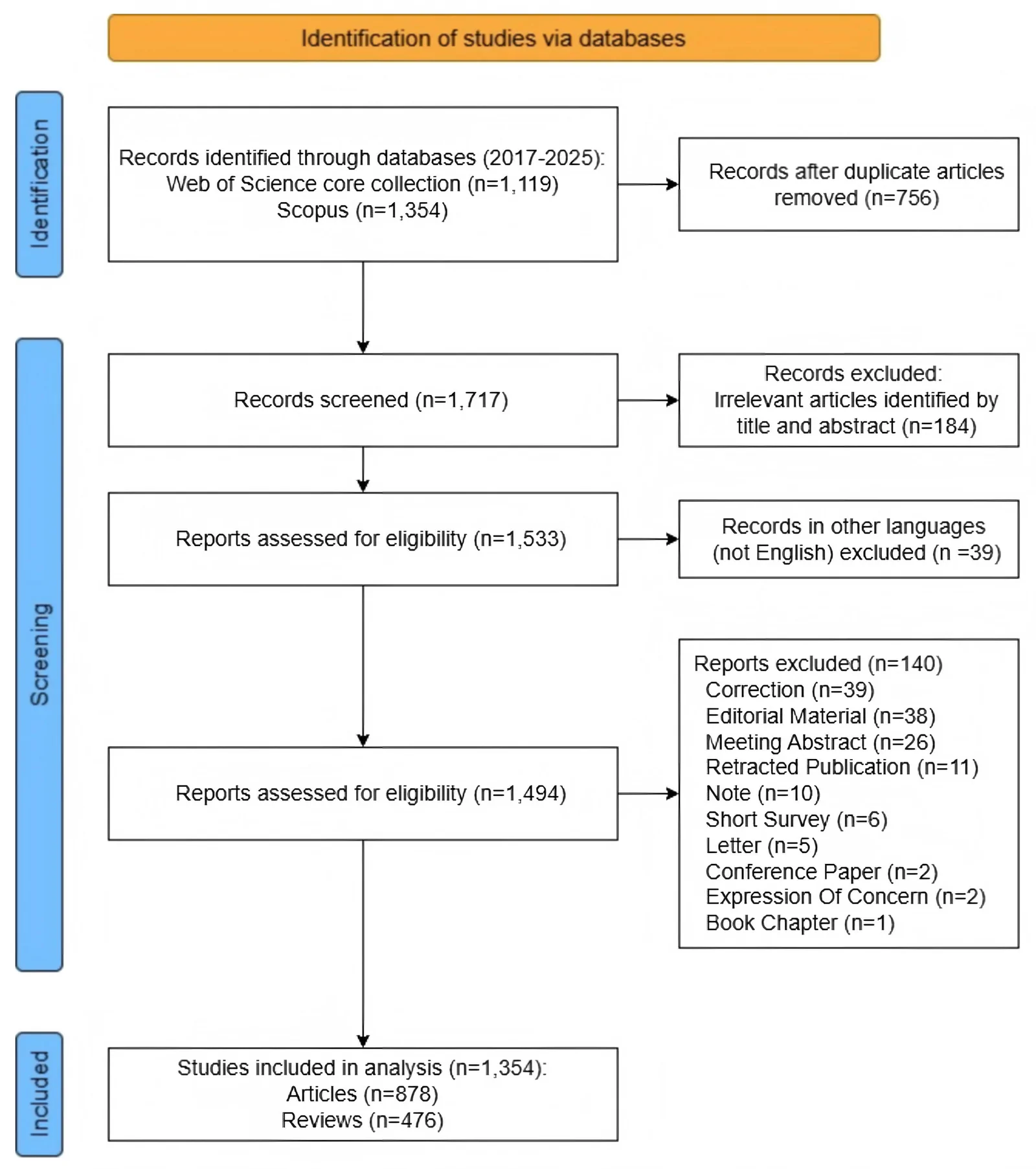
Flowchart of the literature screening process.

### 2.3 Bibliometric and visualization analysis

In this study, VOSviewer (v 1.6.20) (23), CiteSpace (v 6.4.R1) (24), and the R package bibliometrix (version 4.3.3) (25) were used to conduct bibliometric analysis of publications related to ferroptosis and CRC. Data visualization and figure layout were mainly performed using Microsoft Excel 2019 and SCImago Graphica (version 1.0.53). In addition, journal impact factor (IF) information was obtained from the 2025 edition of Journal Citation Reports (JCR).

The bibliometrix package was used to perform systematic bibliometric statistical analyses and to construct and visualize multiple types of networks and matrices. VOSviewer was subsequently used to process large-scale bibliometric data and to construct and analyze knowledge maps of collaboration networks among countries/regions, institutions, authors, and journals, as well as keyword co-occurrence networks. Network associations were normalized using the association strength normalization method. The minimum occurrence threshold for the keyword co-occurrence analysis was set at 30 occurrences, while the minimum publication thresholds for the collaboration analyses of authors, countries/regions, and journals were set at 4, 3, and 5 publications, respectively. CiteSpace was also used to visualize knowledge maps and to identify research hotspots and potential emerging trends through citation analysis and burst detection. Journal dual-map overlay analysis and reference citation burst detection were also performed. The specific parameters were set as follows: the time span was 2017–2025, with a time slice of 1 year; node selection was performed using the g-index (k = 25); keyword clustering was conducted using the log-likelihood ratio (LLR) algorithm; network pruning was performed using the Pathfinder algorithm; and the minimum duration for burst detection was set at 2 years. All other parameters were maintained at their default settings.

## 3 Results

### 3.1 Global publication output and citation trends

From 2017 to 2025, a total of 1, 354 publications on ferroptosis and CRC were published, showing a distinct temporal pattern of academic output (**Figure 3A**). During the early stage from 2017 to 2019, the annual output fluctuated in the single digits. Subsequently, beginning in 2020, the annual number of publications increased sharply, reaching a peak of 385 publications in 2025 and maintaining a high level of research interest. Simple linear regression analysis demonstrated a significant upward trend in annual publication output (B = 53.017 publications/year, 95% CI: 39.626–66.407, *P* < 0.01), with an extremely strong correlation between publication output and time (R^2^ = 0.926). These publications were cited 67, 135 times in the database, and the annual publication output in this field showed a marked inverse trend with the average number of citations per article (**Figure 3B**). From 2017 to 2019, a small number of publications received extremely high average citations, suggesting that these publications constituted the theoretical foundation of this field. The natural decline in average citations per article after 2020 is consistent with the temporal lag in citation accumulation and also indirectly confirms that the field is currently in a highly active developmental stage characterized by rapid iteration of research outputs.

**Figure 3:**
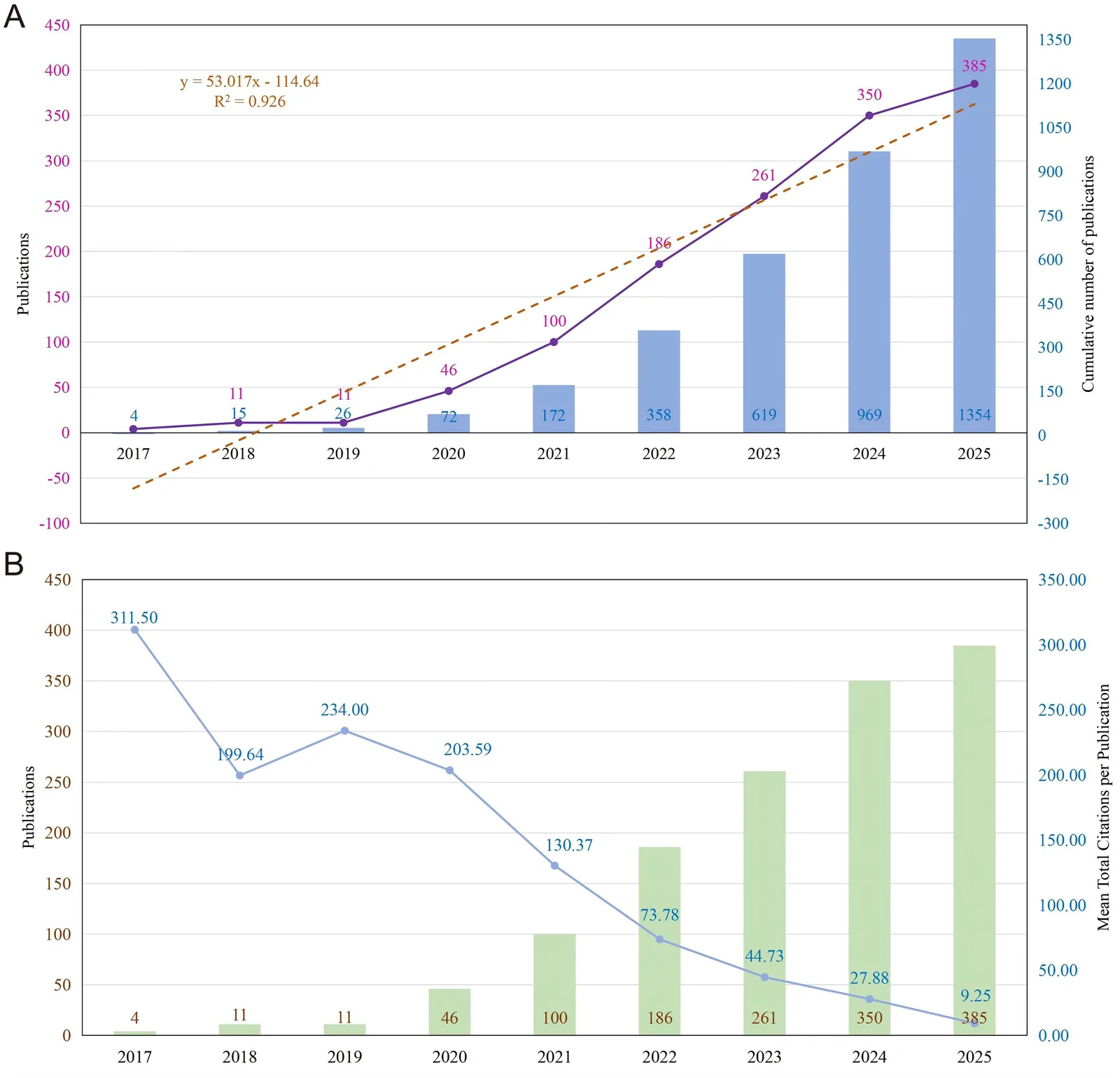
Global publication and citation trends in research on ferroptosis and CRC. **(A)** Annual publication trends. **(B)** Annual trends in the average number of citations per article.

### 3.2 Country/region analysis

Research in this field included contributions from 52 countries (**Figure 4A**), and the geographic distribution of research output showed a global pattern, with a high concentration in Asia, North America, and Europe. **Figure 4B** shows the international collaboration network, in which larger nodes represent greater numbers of publications, and links between countries reflect the intensity of collaboration. The network links indicated that bilateral academic collaboration between China and the United States was the most frequent, with both countries jointly forming the core of international collaboration network. The top 10 countries ranked by total publication output are shown in **Table 1**. China ranked first with 1, 048 publications (75.0%), followed by the United States with 77 publications. China’s high proportion of publications on ferroptosis and CRC may be primarily attributable to its substantial CRC disease burden, continuously increasing investment in biomedical research, and the strong research foundation and broad participation established in studies of ferroptosis and tumor mechanisms. In terms of total citations, China and the United States also ranked highly, with 49, 424 and 6, 501 citations, respectively, whereas most of the other eight countries had fewer than 2, 000 citations. According to the analysis of collaboration types (**Figure 4C**), Australia had a relatively high proportion of multinational collaborative publications (44.4%), indicating its relatively pivotal contribution to this field.

**Figure 4:**
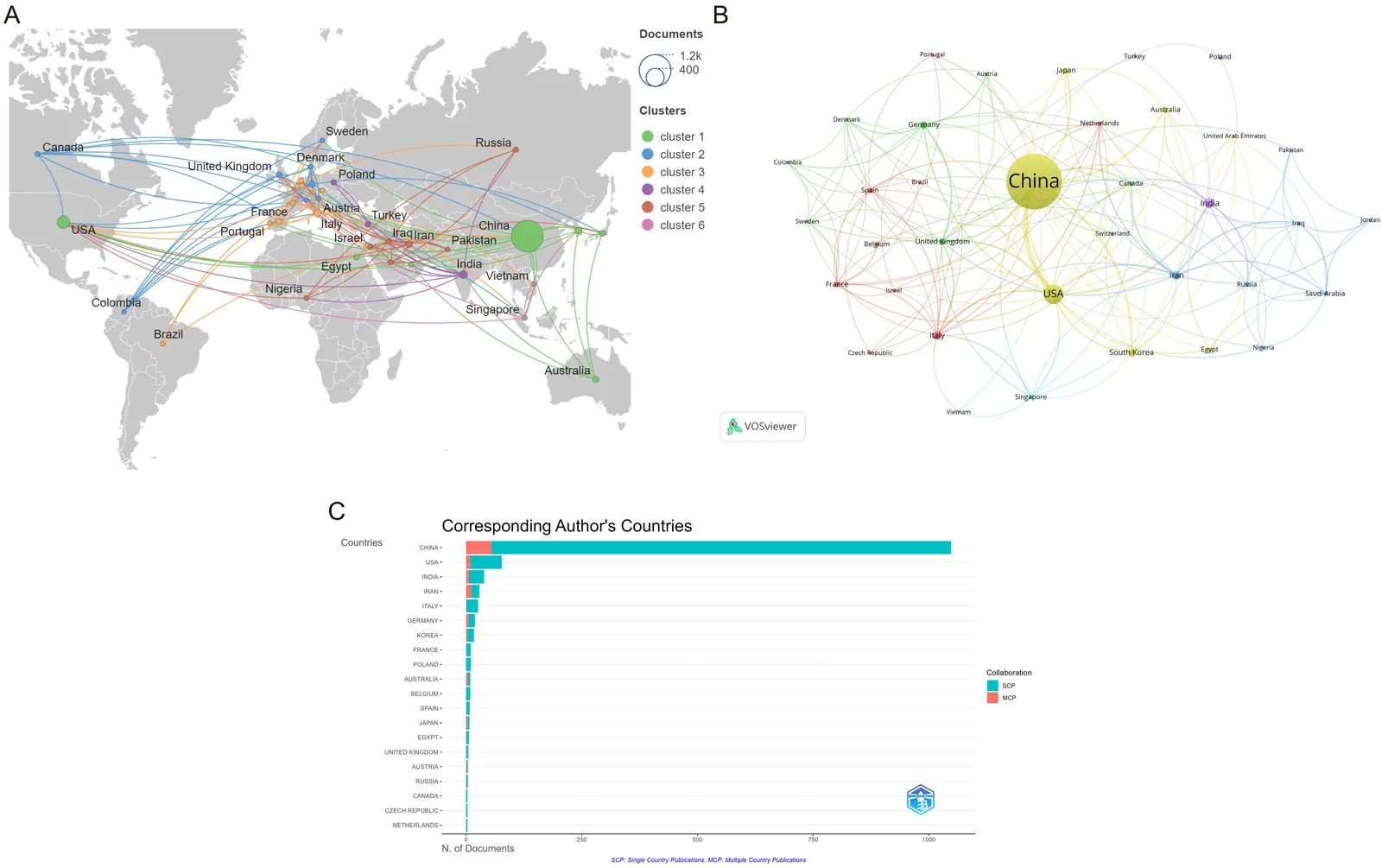
Country-level visualization of publications on ferroptosis in CRC research. **(A)** Global geographic distribution by country/region. **(B)** Collaboration network among different countries. **(C)** Distribution of single-country publications (SCP) and multiple-country publications (MCP) in major countries. The horizontal axis represents the number of publications, and bars in different colors indicate SCP (blue) and MCP (orange), respectively.

**Table 1:** Top 10 countries by publications, SCP, MCP, and citations.

Rank	Country	Publications	SCP	MCP	MCP Ratio	Total citations	Average citations
1	CHINA	1048 (75.0%)	993	55	5.2%	49424	47.2
2	USA	77 (5.5%)	68	9	11.7%	6501	84.4
3	INDIA	39 (2.8%)	33	6	15.4%	1166	29.9
4	IRAN	29 (2.1%)	18	11	37.9%	634	21.9
5	ITALY	26 (1.9%)	25	1	3.8%	823	31.7
6	GERMANY	19 (1.4%)	14	5	26.3%	1044	54.9
7	KOREA	17 (1.2%)	14	3	17.6%	280	16.5
8	FRANCE	10 (0.7%)	9	1	10.0%	928	92.8
9	POLAND	10 (0.7%)	10	0	0.0%	893	89.3
10	AUSTRALIA	9 (0.6%)	5	4	44.4%	575	63.9

### 3.3 Institution and author analysis

A total of 1, 678 research institutions participated in publications in this field, and the top 10 institutions are listed in **Table 2**. Central South University (131 publications) and Fudan University (117 publications) ranked first and second, whereas none of the remaining institutions published more than 100 publications. The institutional collaboration network highlighted close collaborative relationships among Chinese institutions (**Figure 5A**), with Fudan University, Sun Yat-sen University, and Zhejiang University serving as key nodes in the domestic collaboration network. Among institutions outside China, the University of Michigan also demonstrated notable productivity, with 35 publications.

**Table 2:** Most relevant affiliations of the relationship between ferroptosis and CRC.

Rank	Affiliations	Publications
1	Central South University	131
2	Fudan University	117
3	Zhejiang University	98
4	Sun Yat-sen University	97
5	Nanjing Medical University	85
6	Sichuan University	85
7	Shanghai Jiao Tong University	71
8	Jinan University	66
9	Wenzhou Medical University	56
10	Huazhong University of Science and Technology	53

**Figure 5:**
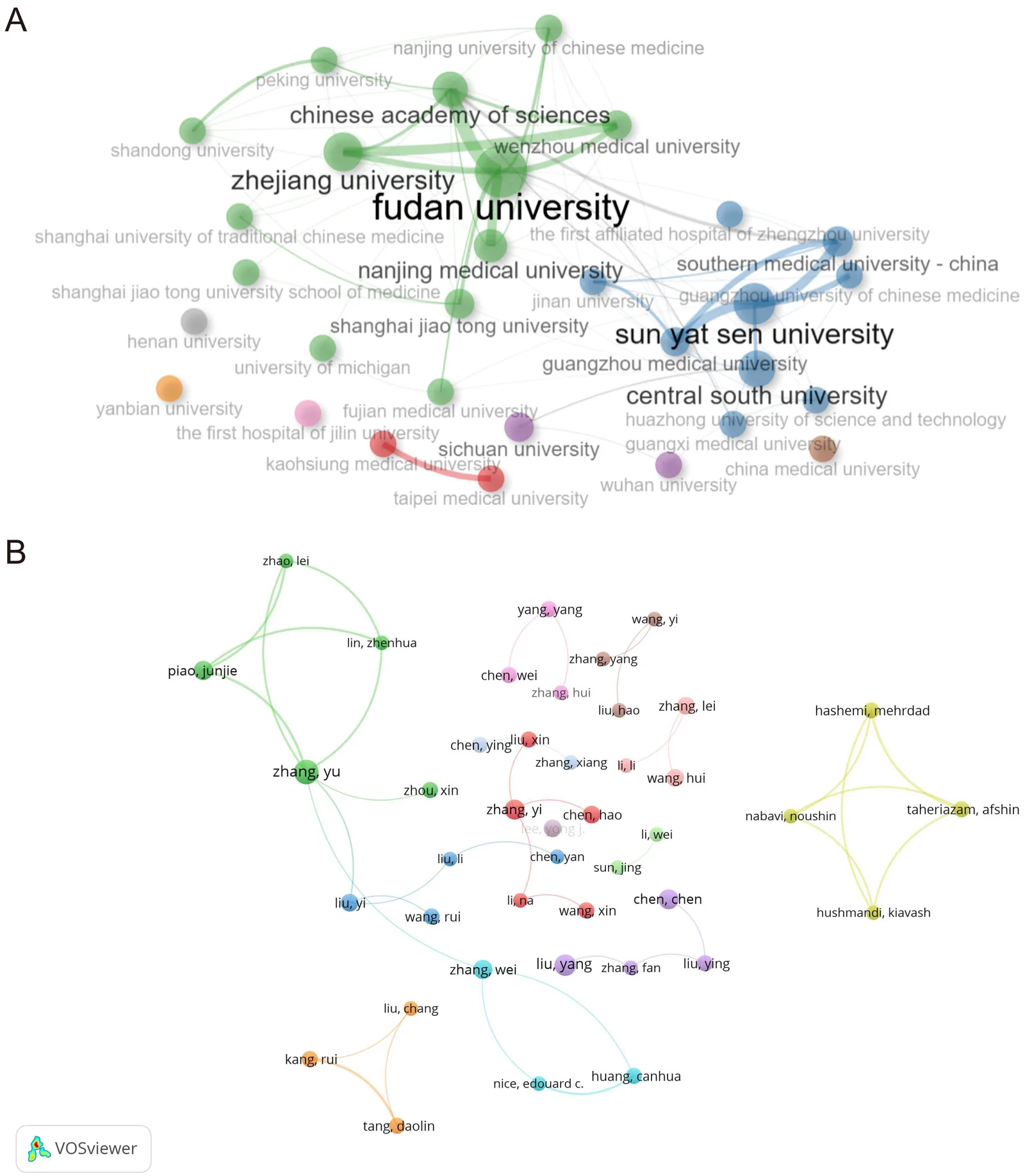
Collaboration networks in research related to ferroptosis and CRC: **(A)** institutional collaboration network; **(B)** author collaboration network.

A total of 9, 531 authors contributed to research on ferroptosis and CRC, forming a collaborative research pattern led by core authors with multinational participation. **Table 3** lists the 10 most productive scholars. Zhang, Yu ranked first with 13 publications, followed by Liu, Yang with 10 publications. These scholars came from three different countries and constituted the core teams driving research in this field. In the author collaboration network (**Figure 5B**), research activity in this field showed a marked pattern of fragmentation. The network consisted of numerous independent subgroups, reflecting close intra-group collaboration, and there is an urgent need to establish broader interdisciplinary and interinstitutional collaborative research systems in the future.

**Table 3:** Top 10 authors of publications and total citations.

Rank	Author	Publications	Cites	Institution	Country
1	Zhang, Yu	13	652	The First Affiliated Hospital of Fujian Medical University	China
2	Liu, Yang	10	667	China-Japan Union Hospital of Jilin University	China
3	Zhang, Yi	9	439	Wannan Medical College	China
4	Zhang, Wei	8	403	Xiangya Hospital, Central South University	China
5	Zhang, Lei	7	453	Henan University	China
6	Chen, Hao	7	373	The First Affiliated Hospital of Wenzhou Medical University	China
7	Chen, Wei	6	208	Guangzhou Medical University	China
8	Hashemi, Mehrdad	6	127	Islamic Azad University	Iran
9	Huang, Canhua	6	508	The First Hospital of Ningbo University	China
10	Kang, Rui	6	1159	UT Southwestern Medical Center	USA

### 3.4 Journal distribution

Research outputs in this field were published in 448 journals and were concentrated in core journals covering multiple disciplines. **Table 4** lists the top 10 journals ranked by publication output. *Frontiers* journals occupied the top three positions, showing a leading trend, followed by *Cancers* with 30 publications, whereas the remaining journals each published fewer than 25 publications. In the VOSviewer analysis, journals with at least five publications were included, and a total of 57 journals were ultimately incorporated into the journal network map (**Figure 6A**). The core clusters showed that the red cluster, centered on Frontiers in Oncology, Frontiers in Pharmacology, and the International Journal of Molecular Sciences, primarily focused on pharmacological mechanisms related to targeted therapeutic interventions for CRC. The green cluster placed greater emphasis on studies involving remodeling of the tumor immune microenvironment, whereas the other clusters further explored fundamental research on redox biology and cell death. The dynamic trends in cumulative publication output of core journals showed that, after 2021, publication output in various oncology and molecular biology journals began to diverge upward collectively, with *Frontiers in Oncology* exhibiting the most rapid growth (**Figure 6B**). The journal dual-map overlay analysis (**Figure 6C**) showed that the research frontiers were mainly concentrated in applied fields such as molecular biology, immunology, and clinical medicine, whereas the knowledge base originated from basic disciplines such as molecular biology and genetics. The core citation pathways demonstrated that current research is highly focused on translating fundamental cellular and genetic mechanisms into frontier applications in tumor immunology and molecular pharmacology.

**Table 4:** Top 10 journals of publications, H-index, IF and JCR.

Rank	Journal	Publications	H-index	IF	JCR
1	Frontiers in Oncology	58	27	3.3	Q2
2	Frontiers in Immunology	47	20	5.9	Q1
3	Frontiers in Pharmacology	39	19	4.8	Q1
4	Cancers	30	20	4.4	Q2
5	International Journal of Molecular Sciences	22	13	4.9	Q1
6	Advanced Science	20	14	14.1	Q1
7	Cell Death & Disease	20	18	9.6	Q1
8	Cells	18	16	5.2	Q2
9	Redox Biology	18	14	11.9	Q1
10	Discover Oncology	16	5	2.9	Q2

*IF, Impact Factor; *JCR, Category Quartile of Journal Citation Report.

**Figure 6:**
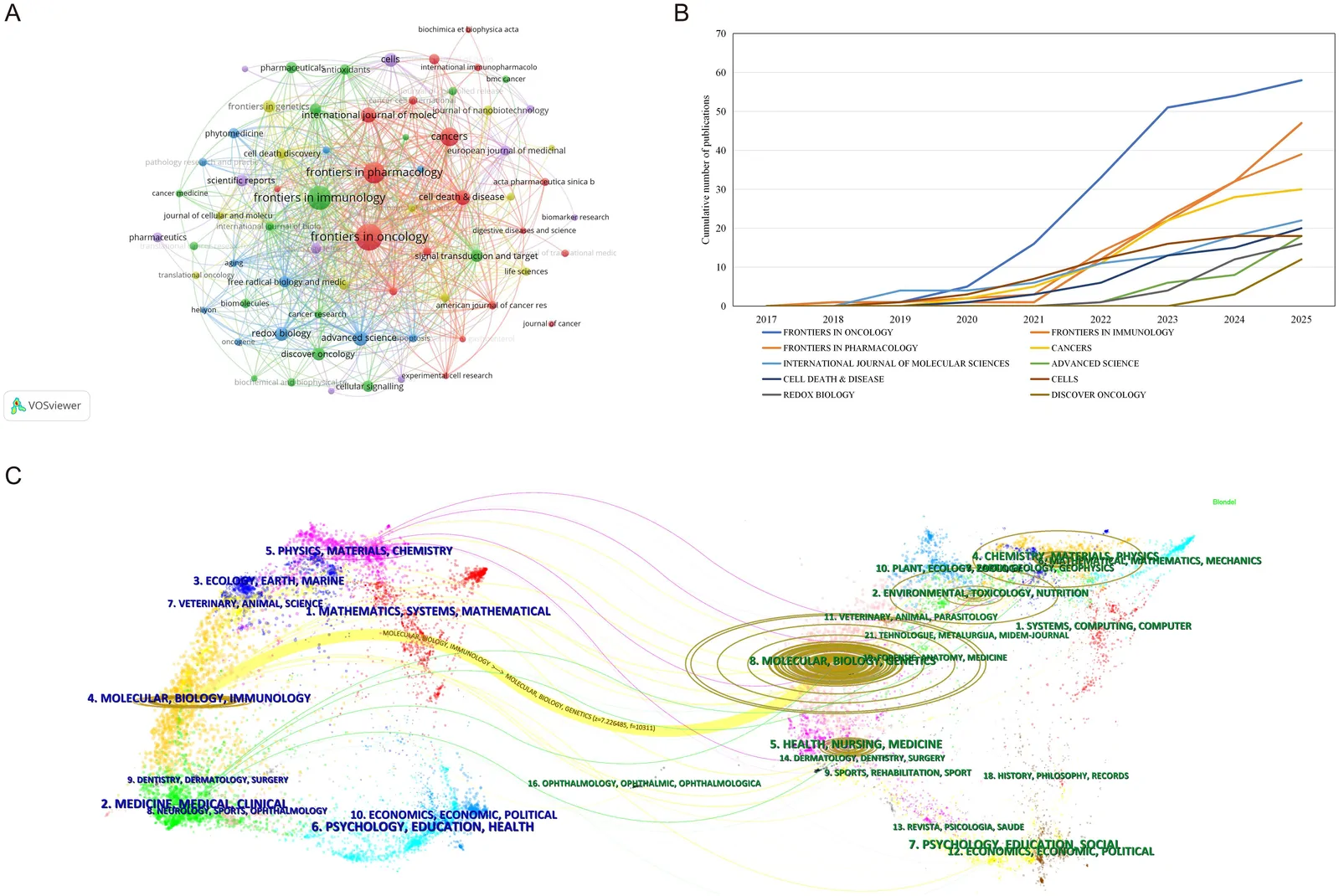
Journal analysis of publications related to ferroptosis in CRC research. **(A)** Journal network map including journals with ≥5 publications. **(B)** Dynamic trends in cumulative publication output of the top 10 journals. **(C)** Journal dual-map overlay analysis.

### 3.5 Analysis of highly cited papers

**Figure 7A** lists the top 10 most cited publications in this field, each of which received more than 700 citations. Among them, the review article published by Li et al. in *Cell Death & Disease* in 2020 ranked first, with a total of 3, 872 citations. This review focused on the progression of ferroptosis and the cellular and molecular mechanisms involved in regulating the pathophysiological processes of multiple diseases, including tumors, neurological diseases, and ischemia-reperfusion injury, emphasizing “intervention in related diseases through regulation of cellular ferroptosis” as a current research hotspot (26). The article published by Koppula et al. in *Protein & Cell* in 2020 (2, 303 citations) and the review published by Mou et al. in *Journal of Hematology & Oncology* in 2019 (1, 748 citations) ranked second and third, respectively. The former revealed the roles of the cystine/glutamate antiporter SLC7A11 in ferroptosis, nutrient dependence, and tumor biology in cancer (27). The latter systematically reviewed aberrant ferroptosis across different cancer types and tissues and integrated research progress on natural compounds as ferroptosis inducers in cancer (28).

**Figure 7:**
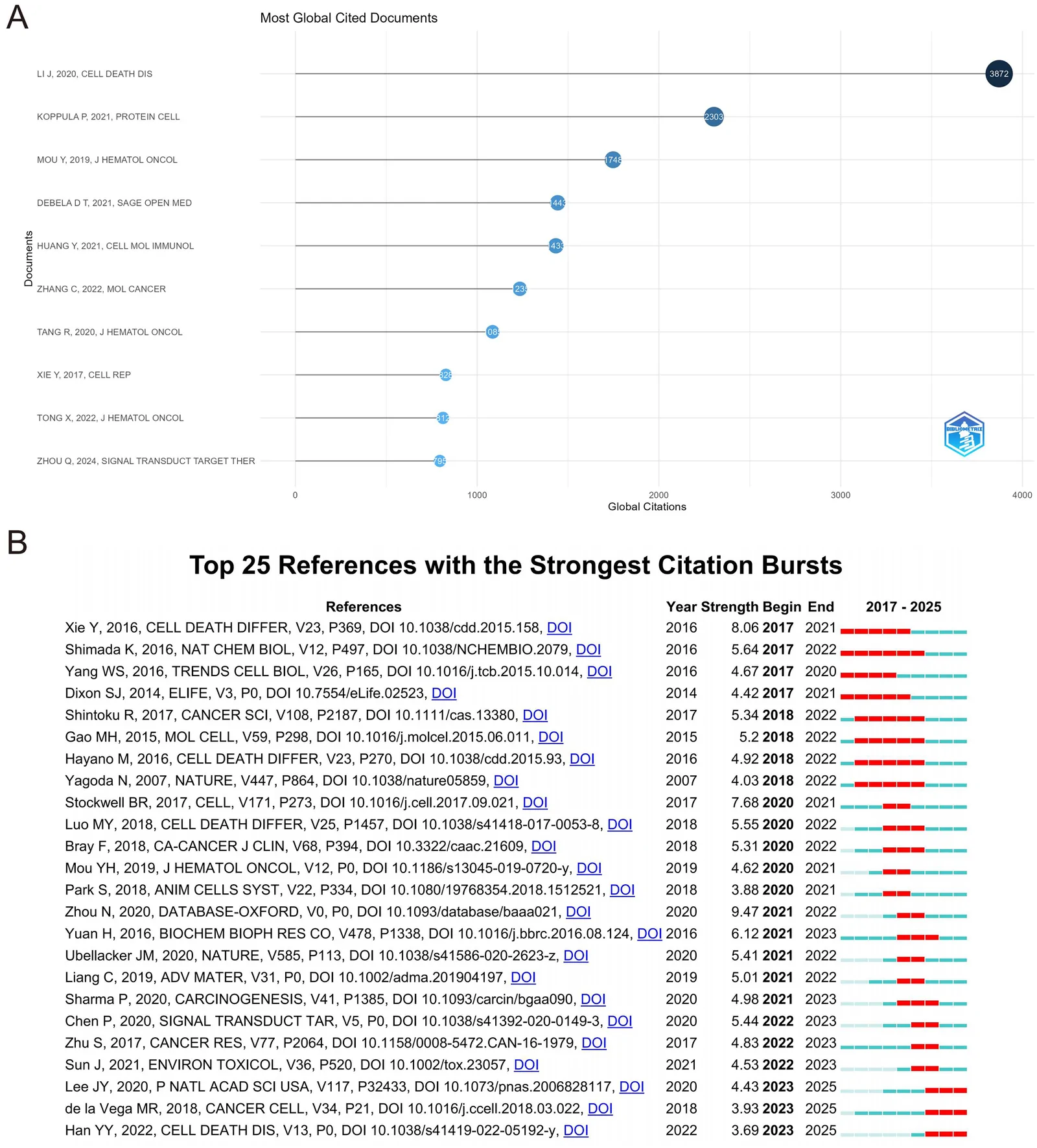
Highly cited publications in ferroptosis research in CRC. **(A)** Citation counts of the top 10 highly cited publications. **(B)** Top 25 references with the strongest citation bursts identified by CiteSpace.

Analysis of citation bursts among co-cited references helps identify key nodes and frontier trends in ferroptosis research in CRC (**Figure 7B**). The red segments in the figure indicate the duration during which specific co-cited references were cited at high frequency, whereas the strength value represents the citation burst intensity of the reference. The article published by Zhou N (2020, Database-Oxford) exhibited the highest burst strength of 9.47 from 2021 to 2022. This study established FerrDb, the first database dedicated to ferroptosis, to provide information on ferroptosis and factors associated with related diseases. In addition, the study by Stockwell BR (2017, Cell) showed the burst intensity from 2020 to 2021, with a burst strength of 7.68, and systematically established the metabolic network basis of ferroptosis and its tumor-suppressive function. Notably, the study by Yagoda N (2007, Nature) first discovered that the classical inducer erastin could trigger a non-apoptotic form of cell death. With the deepening of ferroptosis research in oncology from 2018 to 2022, this foundational publication suddenly gained prominence after remaining dormant for a decade. Studies by Lee JY (2020, PNAS), de la Vega MR (2018, Cancer Cell), and Han YY (2022, Cell Death & Disease) continued to show citation bursts until 2025, representing the most active current research frontiers. Research is no longer limited to the classical System Xc^-^/GPX4 axis but has extended into noncanonical metabolic pathways, regulation of the hypoxic microenvironment, and crosstalk with other modes of cell death.

### 3.6 Keyword analysis

Keywords are commonly used to highlight popular research directions and summarize the content of publications. By analyzing keyword co-occurrence frequency relationships and burst detection, research hotspots and trends in this field can be accurately identified (29). In this study, a keyword heatmap was constructed using VOSviewer (**Figure 8A**). Ferroptosis and CRC were the core themes in this field. Peripheral keywords, including lipid peroxidation, iron, metabolism, apoptosis, and reactive oxygen metabolites, reflected investigations into the fundamental mechanisms of ferroptosis. Keywords such as tumor microenvironment, signal transduction, gene expression, and tumor growth indicated that research had further expanded to examine the roles of ferroptosis in CRC initiation and progression, the tumor microenvironment, and molecular signaling regulation. In addition, treatment-related keywords, including oxaliplatin, fluorouracil, cisplatin, drug resistance, and chemotherapy, were also identified, indicating that the relationship between ferroptosis and chemosensitivity, as well as the mechanisms of chemoresistance, represents another important research direction in this field. In the dynamic thematic evolution map of this field (**Figure 8B**), the results revealed an evolutionary pattern from basic mechanistic exploration toward translational targeted therapy. Since 2020, with the burst emergence of ferroptosis-related molecular markers such as CD71 antigen and heme oxygenase-1, research from 2022 to 2024 rapidly shifted toward metabolic reprogramming of ferroptosis and the mechanisms underlying apoptosis induction in CRC models. During 2025, gut microbiota and induced apoptosis rapidly emerged as core hotspots, indicating that the gut microbiome and target receptor-related mechanisms may represent current research frontiers in this field.

**Figure 8:**
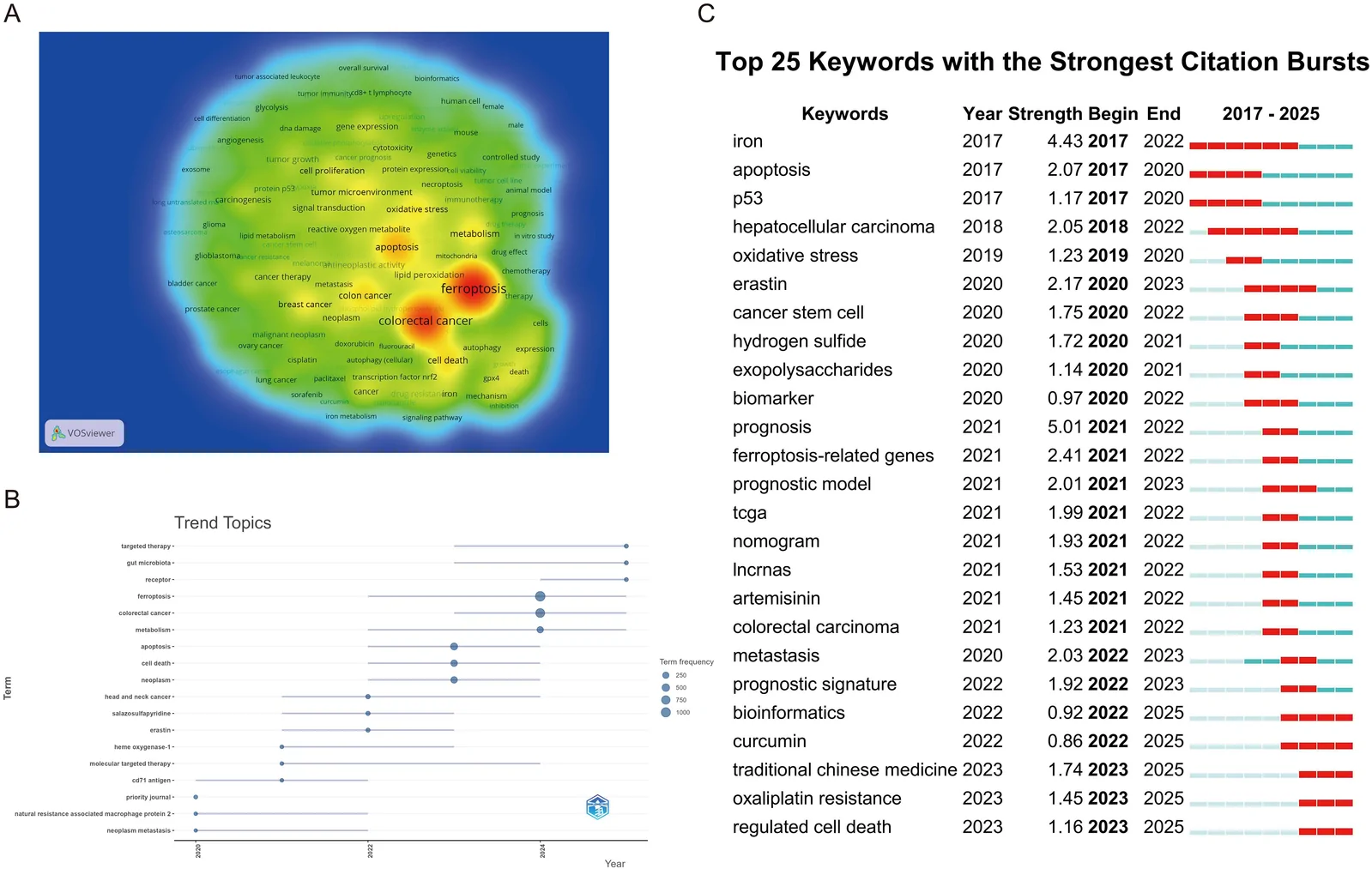
Keyword analysis of ferroptosis in CRC research. **(A)** Keyword heatmap generated by VOSviewer. **(B)** Thematic trend analysis based on keywords. **(C)** Top 25 keywords with the highest burst strength, generated by CiteSpace.

Keyword burst detection is an effective method for tracking the evolution of research hotspots within specific time periods (30). Through keyword burst analysis, the top 25 keywords with the strongest burst intensity were identified (**Figure 8C**). From 2017 to 2020, research mainly focused on classical tumor suppressor genes such as p53 and the macroscopic mechanisms of cellular carcinogenesis. From 2020 to 2023, “prognosis” reached the highest burst intensity, reflecting increased attention to disease prognosis and treatment outcomes. During this period, keywords such as “prognostic model,” “nomogram,” and “ferroptosis-related genes” became prominent, indicating that bioinformatics and prognostic analysis became rapidly emerging hotspots. Of particular note, from 2023 to 2025, keywords such as “oxaliplatin resistance,” “traditional Chinese medicine,” “bioinformatics,” “curcumin,” and “regulated cell death” showed sustained strong bursts. This analysis demonstrates the transition of the field from bioinformatic prediction to clinical translational medicine, with in-depth exploration of how traditional Chinese medicine and natural products reverse chemotherapy resistance in CRC by regulating cell death networks, constituting one of the most promising current research directions.

## 4 Discussion

### 4.1 Overview

This study analyzed and interpreted 1, 354 publications on this topic to identify key research hotspots and trends. These publications were published in 448 journals by 9, 531 authors from 1, 678 institutions across 52 countries. Based on studies of erastin-induced death in RAS-mutant cancer cells, Brent R. Stockwell’s team first proposed the term “ferroptosis” in 2012 to describe a novel iron-dependent form of regulated cell death that is distinct from apoptosis, autophagy, and necrosis (31). Over the past nine years, academic interest and research activity regarding ferroptosis in CRC have increased markedly, resulting in steady annual growth in the number of related publications. This trend suggests that this research field will continue to receive extensive attention. In the ranking of publication output in this field, China produced a significantly higher number of publications. Currently, China and the United States are leading in this field, and most of the top 10 research institutions and scholars are from China. Among them, Central South University made particularly prominent contributions, and Zhang, Yu, the most productive author, was the only author with 13 publications. The formation of current research trends may be attributable to several factors, including the high incidence of CRC in the Chinese population, the relatively high prevalence of iron metabolism-related diseases, and the unique research direction pursued by Chinese researchers in cancer therapy through the use of traditional Chinese medicine and natural products to induce ferroptosis. Analysis of publication journals showed that more than one-fifth of the publications were published in the top 10 journals, among which Frontiers in Oncology and Frontiers in Immunology made substantial contributions to this field. These journals have broadly disseminated studies on the role of ferroptosis in CRC, covering molecular regulatory mechanisms, construction of prognostic prediction models, regulation of chemotherapy resistance, the tumor immune microenvironment, interventions using natural products and traditional Chinese medicine, and nanodrug delivery systems. These contributions have substantially advanced researchers’ understanding of this field.

### 4.2 Hotspots and development trends

The thematic hotspots discussed below were directly derived from the bibliometric findings, particularly the co-cited references and keyword burst analyses with continuing influence shown in **Figures 7** and **8**. Burst emergence indicates a significant increase in keyword usage over a short period, reflecting the rapid rise in prominence of terms that are critical for defining the field. These structures reveal how the field has evolved from mechanistic research to translational applications.

#### 4.2.1 Role of ferroptosis in chemotherapy resistance of CRC cells

Chemotherapy resistance leads to treatment failure in some patients, and targeted activation of ferroptosis can overcome acquired drug resistance (31), with the potential to improve sensitivity to chemotherapeutic agents such as oxaliplatin, 5-fluorouracil, and irinotecan. In chemotherapy resistance, resistant cells usually enhance their antioxidant and anti-ferroptotic capacities, making the ROS generation and lipid peroxidation induced by chemotherapy insufficient to reach a lethal threshold. For instance, inhibition of the KIF20A/NUAK1/Nrf2/GPX4 pathway can induce ferroptosis and enhance the sensitivity of CRC cells to oxaliplatin (32). Another study using a Clustered Regularly Interspaced Short Palindromic Repeats library screen revealed that NFS1 deficiency synergizes with oxaliplatin to increase intracellular ROS levels in CRC cells and trigger PANoptosis, including ferroptosis, thereby enhancing oxaliplatin sensitivity (33). Meanwhile, ferroptosis requires membrane phospholipids containing polyunsaturated fatty acids as oxidative substrates, and ACSL4 is an important factor that promotes the incorporation of these lipids into membrane phospholipids. Related studies have found that inhibition of CDK1 can overcome oxaliplatin resistance by regulating ACSL4-mediated ferroptosis (34). In addition, aberrant activation of the Nrf2 signaling pathway is also involved in multidrug resistance. Studies have shown that high CBX3 expression is associated with post-chemotherapy progression and poorer survival; CBX3 can inhibit ferroptosis through the CUL3/NRF2/GPX2 axis and enhance CRC resistance to irinotecan and oxaliplatin, whereas ferroptosis activators such as RSL3 and FIN56 can attenuate the CBX3-mediated resistant phenotype (35). Regulation of intracellular iron homeostasis also affects resistant cells. Studies have found that targeting the FBXL5/IREB2/TFRC axis can disrupt iron homeostasis in oxaliplatin-resistant CRC cells. FBXL5 knockdown upregulates IREB2/TFRC, increases Fe²^+^ and MDA levels, and enhances oxaliplatin-induced cytotoxicity, whereas the ferroptosis inhibitor ferrostatin-1 reverses this effect (36).

#### 4.2.2 Ferroptosis enhances immunotherapy, regulates the tumor microenvironment, and crosstalks with other modes of cell death in the context of CRC

Current research on ferroptosis in CRC has expanded from the early classical framework centered on iron metabolism, lipid peroxidation, and the SLC7A11/GSH/GPX4 signaling axis to a complex network encompassing immune regulation and tumor microenvironment (TME) remodeling. Ferroptosis is considered one of the important effector mechanisms of cancer immunotherapy; immunotherapy-activated CD8^+^ T cells can promote ferroptosis-specific lipid peroxidation in tumor cells and reduce their cystine uptake, thereby enhancing the antitumor effects of immunotherapy (37). In addition, ferroptosis not only acts directly on tumor cells but also indirectly improves immunotherapeutic efficacy by regulating immune cell activity and the state of the TME (38). In CRC, the TME is enriched with immunosuppressive cells, such as tumor-associated macrophages and regulatory T cells, which secrete inhibitory cytokines and express immune checkpoint molecules such as PD-L1, thereby suppressing T-cell activity and evading immune surveillance (39). Ferroptosis can markedly affect immune homeostasis in the TME by regulating immune cell function (40). For example, ferroptosis can remodel macrophage phenotypes in the TME and promote the conversion of M2 macrophages toward an antitumor M1 phenotype, thereby enhancing antitumor immune responses (41). Therefore, the combined application of ferroptosis inducers and immunotherapy may constitute a promising therapeutic strategy for CRC. This strategy can not only directly kill tumor cells but also activate the immune system by inducing immunogenic cell death, thereby promoting tumor-specific immune responses (42).

Functional crosstalk exists between ferroptosis and pyroptosis, necroptosis, and apoptosis. Studies have shown that NFS1 deficiency synergizes with oxaliplatin to enhance oxaliplatin sensitivity in CRC cells by increasing intracellular ROS levels and triggering PANoptosis, a composite cell death program involving apoptosis, necroptosis, pyroptosis, and ferroptosis (33). In addition, Nrf2 inhibition can increase CRC sensitivity to oxaliplatin by simultaneously promoting ferroptosis and pyroptosis, suggesting that once the antioxidant transcriptional program is relieved, cells may enter a state in which multiple death pathways proceed in parallel rather than undergoing ferroptosis alone (43).

#### 4.2.3 Development of prognostic models and biomarkers related to ferroptosis in CRC therapy

Expression patterns of ferroptosis-related genes provide new prognostic tools for CRC therapy and are of great significance for personalized medicine and precision treatment. FerrDb is one of the most commonly used data sources for this type of research, and FerrDb V2 includes 1, 001 ferroptosis regulators and 143 ferroptosis-disease association expression patterns (44). Bioinformatics analyses of The Cancer Genome Atlas (TCGA) and Gene Expression Omnibus (GEO) databases have enabled the construction of prognostic models based on ferroptosis-related genes (FRGs), facilitating patient risk stratification and therapeutic guidance. One study developed an 11-gene signature through WGCNA, univariate Cox regression, and Lasso Cox regression, consisting of CD1B, ATP6V1B2, SLC2A3, TMC8, CD36, HDC, SHISAL2A, MFNG, TOX2, DAPK1, and CCL22, which could effectively predict overall survival in patients with CRC (45). In addition, Nie et al. established a typical mRNA-based multigene prognostic risk model containing five CRC- and ferroptosis-related genes, namely AKR1C1, ALOX12, FDFT1, ATP5MC3, and CARS1. The 1-, 2-, and 3-year AUC values of this model were approximately 0.668, 0.678, and 0.686, respectively, and the model suggested different immune states between high- and low-risk groups, particularly involving CD8^+^ T cells and STING-related signaling (46).

Ferroptosis-related genes and noncoding RNAs also provide a strong foundation for the development of clinical biomarkers for diagnosis, prognostic evaluation, and treatment monitoring. lncRNAs, such as Lnc-LSS-1 and Lnc-PEX11G-4, have been found to inhibit RSL3- and erastin-induced ferroptosis after knockdown, involving lipid peroxidation and GPX4 regulation (47). Ferroptosis-related prognostic models in CRC serve as complementary tools for identifying patients at high risk of recurrence, predicting the benefits of adjuvant chemotherapy, explaining immunotherapy tolerance, and screening populations suitable for pro-ferroptotic combination therapy, thereby revealing the value of these models in precision immuno-oncology.

#### 4.2.4 Intervention of the gut microbiota in ferroptosis in CRC

The gut microbiota can both enhance ferroptosis in CRC cells and help tumor cells resist ferroptosis. High abundance of Fusobacterium nucleatum in CRC tissues is associated with recurrence. Studies have found that F. nucleatum can inhibit ferroptosis through the E-cadherin/β-catenin/GPX4 axis and promote oxaliplatin resistance (48). In addition, Peptostreptococcus anaerobius and its tryptophan metabolite trans-3-indoleacrylic acid (IDA) can inhibit ferroptosis and promote CRC initiation and progression through the AHR–ALDH1A3–NADH–FSP1–CoQ10 axis (49). Meanwhile, gut microbiota metabolism is closely associated with CRC progression, and microbial-derived metabolites can affect tumor plasticity through ferroptosis. Targeting gut microbiota-mediated ferroptosis may become a therapeutic strategy for CRC (50). Studies have shown that butyrate levels are decreased in patients with CRC. Butyrate can inhibit xCT/SLC7A11 by inducing c-Fos, reduce xCT-dependent GSH synthesis, decrease ferroptosis resistance in cancer stem cells, and enhance sensitivity to ferroptosis induction in animal models (51).

#### 4.2.5 Regulatory mechanisms of natural compounds from traditional chinese medicine in ferroptosis in CRC

Traditional Chinese medicine (TCM) has demonstrated substantial potential in oncology because of its multitarget and multipathway mechanisms. Active molecules in TCM can inhibit tumor growth, reverse chemotherapy resistance, and potentially enhance immunotherapy by inducing iron-dependent lipid peroxidation in CRC cells. Baicalein induces ferroptosis in CRC cells by directly interacting with JAK2 and blocking the JAK2/STAT3/GPX4 axis (52). Unlike conventional therapies that focus on single-target intervention in tumor cells, TCM has expanded toward dual-target or multitarget ferroptosis-regulatory networks. For example, combined treatment of CRC cells and patient-derived organoids with curcumin and Andrographis extract can simultaneously downregulate GPX4 and FSP1 and increase ROS and lipid peroxidation, thereby exerting antitumor effects (53). In addition, β-elemene is a novel ferroptosis inducer, and its combination with cetuximab can induce ferroptosis in KRAS-mutant CRC cells and inhibit epithelial-mesenchymal transition, demonstrating the effect of TCM in reversing therapeutic resistance and enhancing combination therapy (54). TCM intervenes in CRC progression by regulating the tumor immune microenvironment and gut microbiota-mediated ferroptosis. Studies have shown that fecal microbiota from curcumin-treated mice can slow CRC tumor growth, enhance CD8^+^ T-cell tumor infiltration, and induce ferroptosis in tumor cells; after antibiotic-mediated depletion of the microbiota, the antitumor effect of curcumin disappears, suggesting that its effect is gut microbiota-dependent (55). In addition, icariin has been reported to promote ferroptosis in CRC cells by inducing mitochondrial dysfunction and to exert synergistic antitumor effects with PD-1 inhibitors (56). These studies highlight the multidimensional therapeutic approaches of natural compounds from TCM in inducing ferroptosis and regulating immune responses, representing a promising direction for advancing CRC treatment.

### 4.3 Limitations

This study used literature indexed in the WoSCC and Scopus databases as data sources, conducted a systematic bibliometric analysis, and used visualization software to predict future research hotspots and trends. Nevertheless, this study has several limitations. First, geoeconomic differences among countries may lead to systematic bias in research productivity and representativeness. Second, restriction to English-language publications in WoSCC and Scopus may introduce bias and thereby limit the generalizability of the results; therefore, future studies should integrate multilingual source databases such as CNKI and PubMed. Finally, owing to the inherent citation delay of the literature, related studies usually require 3–10 years to reach a citation peak, which may obscure the impact of recent high-quality publications. Although these limitations restrict temporal and geographic generalizability, this systematic approach can still lay a foundation for subsequent research.

From the perspective of clinical translation, incorporating ferroptosis-regulatory strategies into the CRC treatment system has substantial potential, particularly in providing new insights for improving therapeutic responses, overcoming drug resistance, and optimizing combination treatment regimens. However, this field still faces several key challenges, including the targeting specificity and safety evaluation of ferroptosis inducers, as well as further clarification of different resistance mechanisms. Future research should focus on optimizing combination treatment strategies, screening potential biomarkers, and conducting prospective clinical trials to validate the practical application value of basic research findings in the clinical management of CRC.

## 5 Conclusion

This study systematically analyzed the research trends, knowledge structure, and frontier hotspots in ferroptosis and CRC from 2017 to 2025 using bibliometric methods. Currently, the relationship between ferroptosis and CRC has become an important and rapidly developing direction in global oncology research, with related research output showing a marked increase after 2020. China and the United States occupy dominant positions in this field, with China showing particularly prominent performance in both publication output and total citations. In this field, Frontiers in Oncology and Frontiers in Immunology have been established as key publication platforms. Current research hotspots focus on the mechanistic role of ferroptosis in chemotherapy resistance in CRC, as well as potential strategies for enhancing sensitivity to chemotherapeutic agents by inducing ferroptosis. Meanwhile, the research focus is gradually expanding from the classical SLC7A11/GSH/GPX4 axis, iron homeostasis imbalance, and lipid peroxidation mechanisms to precision medicine directions, including remodeling of the tumor immune microenvironment, sensitization to immunotherapy, crosstalk among cell death modalities such as PANoptosis, non-coding RNAs, ferroptosis-related gene signatures, and prognostic model construction. Regulation of ferroptosis by the gut microbiota, traditional Chinese medicine, and natural products is also emerging as a new frontier with translational potential in CRC therapy. Incorporating ferroptosis-related molecular features, immune infiltration patterns, and gut microbiota status into precision treatment systems for CRC may become an important direction for promoting continuous innovation and clinical translation in this field.

## Data Availability

Publicly available datasets were analyzed in this study. This data can be found here: Web of Science database and Scopus database: Direct link: https://www.webofscience.com; https://www.scopus.com.
